# 9-(2-Pyridylmeth­oxy)-1,10-phenanthrolin-1-ium perchlorate methanol solvate

**DOI:** 10.1107/S1600536808020606

**Published:** 2008-07-09

**Authors:** Shi Guo Zhang, Chao Hou

**Affiliations:** a Department of Chemistry and Chemical Engineering, Institute of Materials Chemistry, Binzhou University, Binzhou 256603, People’s Republic of China; b Department of Chemistry, Shandong Normal University, Jinan 250014, People’s Republic of China

## Abstract

In the title organic salt, C_18_H_14_N_3_O^+^·ClO_4_^−^·CH_4_O, there is a π–π stacking inter­action between neighbouring 1,10-phenanthroline rings and the relevant distances are 3.5453 (18) Å for the centroid–centroid distance and 3.354 Å for the perpendicular distance. There is also a relatively close contact between a C—H bond and a symmetry-related pyridine ring. There are classical N—H⋯O and O—H⋯N hydrogen bonds and non-classical C—H⋯O hydrogen bonds involving the cation, methanol solvent mol­ecule and perchlorate anion.

## Related literature

For a related structure, see: Liu *et al.* (2008 ▶).
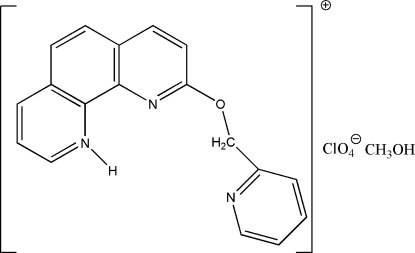


## Experimental

### 

#### Crystal data

C_18_H_14_N_3_O^+^·ClO_4_^−^·CH_4_O
*M* *_r_* = 419.81
Triclinic, 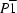

*a* = 7.0765 (15) Å
*b* = 10.597 (2) Å
*c* = 14.164 (3) Å
α = 110.003 (3)°
β = 94.999 (3)°
γ = 105.304 (3)°
*V* = 944.1 (3) Å^3^
*Z* = 2
Mo *K*α radiation
μ = 0.25 mm^−1^
*T* = 298 (2) K
0.38 × 0.31 × 0.18 mm

#### Data collection

Bruker SMART APEX CCD diffractometer
Absorption correction: multi-scan (*SADABS*; Sheldrick, 1996 ▶) *T*_min_ = 0.912, *T*_max_ = 0.957
5027 measured reflections
3504 independent reflections
2695 reflections with *I* > 2σ(*I*)
*R*_int_ = 0.019

#### Refinement

*R*[*F* ^2^ > 2σ(*F* ^2^)] = 0.057
*wR*(*F* ^2^) = 0.164
*S* = 1.05
3504 reflections
263 parameters
H-atom parameters constrained
Δρ_max_ = 0.41 e Å^−3^
Δρ_min_ = −0.23 e Å^−3^

### 

Data collection: *SMART* (Bruker, 1997 ▶); cell refinement: *SAINT* (Bruker, 1997 ▶); data reduction: *SAINT*; program(s) used to solve structure: *SHELXTL* (Sheldrick, 2008 ▶); program(s) used to refine structure: *SHELXTL*; molecular graphics: *SHELXTL*; software used to prepare material for publication: *SHELXTL* and local programs.

## Supplementary Material

Crystal structure: contains datablocks I, global. DOI: 10.1107/S1600536808020606/bq2085sup1.cif

Structure factors: contains datablocks I. DOI: 10.1107/S1600536808020606/bq2085Isup2.hkl

Additional supplementary materials:  crystallographic information; 3D view; checkCIF report

## Figures and Tables

**Table 1:** Hydrogen-bond geometry (Å, °)

*D*—H⋯*A*	*D*—H	H⋯*A*	*D*⋯*A*	*D*—H⋯*A*
N1—H4⋯O6	0.81	1.85	2.648 (3)	167
O6—H5⋯N3	0.89	1.85	2.738 (4)	175
C3—H3⋯O2	0.93	2.32	3.238 (5)	170
C13—H13*B*⋯O6	0.97	2.58	3.405 (4)	143
C8—H8⋯*Cg*3^i^	0.93	2.81	3.650 (2)	151

Symmetry code: (i) 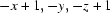. *Cg*3 is the centroid of the pyridine ring

**Fig. 1.**
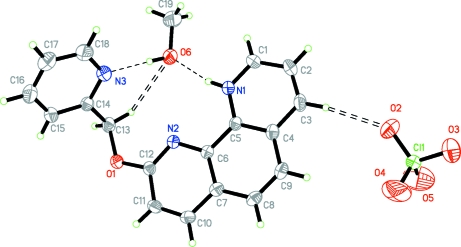
Structure of title organic salt with the atom-numbering scheme. Displacement ellipsoids are drawn at the 30% probability level. The classic hydrogen bonds are shown as dashed lines and non-classic hydrogen bonds as double dashed lines.

**Fig. 2.**
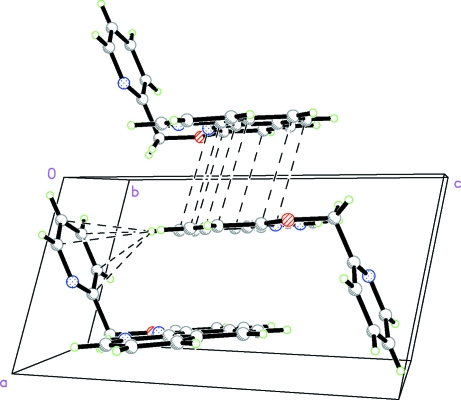
The packing and π-π stacking interaction (dashed lines) and C—H···π interaction (dashed lines), the methanol molecule and perchlorate anion have been omitted for clarity.

## supplementary crystallographic information

### Comment

Derivatives of 1,10-phenanthroline play an important role in modern coordination chemistry (Liu *et al.*,  2008) and we have tried to prepare a complex containing Manganese(II) and iron(III) metallic ions and 2-((pyridin-2-yl)methoxy)-1,10-phenanthroline ligand, but we obtained the title organic salt.

Fig. 1 shows the structure, revealing that one of N atoms from phenanthroline ring was protonated and it was turned into a cation. There is a π-π stacking interaction involving symmetry-related 1,10-phenanthroline rings, the relevant distances being *Cg*1···*Cg*2^i^ = 3.5453 (18) Å and *Cg*1···*Cg*2^i^_perp_ = 3.354 Å and α = 1.09°; there also exits interaction between C8-H8 bond and pyridine ring and the relevant distance is H8···*Cg*3^ii^ = 2.81 Å for H8 atom to the centroid of the pyridine ring and H8···*Cg*3^ii^_perp_ = 2.803 Å for the perpendicular distance from H8 atom to the pyridine ring plane [symmetry code: (i) -*X*, -*Y*, 1-*Z*; (ii) 1-*X*, -*Y*, 1-*Z*; *Cg*1, *Cg*2 and *Cg*3 are the centroids of the N2/C6C7C10-C12 ring, C4-C9 ring and N3/C14-C18 rings, respectively; *Cg*1···*Cg*2^i^_perp_ is the perpendicular distance from ring *Cg*1 to ring *Cg*2^i^; α is the dihedral angle between ring plane *Cg*1 and ring plane *Cg*2^i^; ]. There exist N-H···O and O-H···N classic hydrogen bonds and C-H···O non-classic hydrogen bonds (Fig. 1 and Table 1) in the asymmetric unit.

### Experimental

10 ml methanol solution of 2-((pyridin-2-yl)methoxy)-1,10-phenanthroline (0.1200 g, 0.418 mmol) was added into 20 ml methanol solution containing FeCl_3_.6H_2_O (0.0565 g, 0.209 mmol) and Mn(ClO_4_)_2_.6H_2_O (0.0757 g, 0.209 mmol), and the mixed solution was stirred for half a hour. Yellow single crystals were obtained after the solution had been allowed to stand at room temperature for three days.

### Refinement

Nitrigen-bound H atom and Oxygen-bound H atom were located in a difference Fourier map, then placed in calculated positions with N—H = 0.81 Å and O—H = 0.89 Å and refined as riding with *U*_iso_(H) = 1.2*U*_eq_(N) and *U*_iso_(H) = 1.5*U*_eq_(O). Other H atoms were placed in calculated positions with with C—H = 0.97 Å for methyl, C—H = 0.96 Å for methylene and C—H = 0.93 Å for other H atoms, and refined as riding with *U*_iso_ = 1.5*U*_eq_(C) for methyl H and *U*_iso_ = 1.2*U*_eq_(C) for other H atoms.

### Crystal data

**Table:**

C_18_H_14_N_3_O^+^·ClO_4_^–^·CH_4_O	*Z* = 2
*M**_r_* = 419.81	*F*_000_ = 436
Triclinic, *P*1	*D*_x_ = 1.477 Mg m^−^^3^
Hall symbol: -P 1	Mo *K*α radiation λ = 0.71073 Å
*a* = 7.0765 (15) Å	Cell parameters from 1588 reflections
*b* = 10.597 (2) Å	θ = 3.0–25.9º
*c* = 14.164 (3) Å	µ = 0.25 mm^−^^1^
α = 110.003 (3)º	*T* = 298 (2) K
β = 94.999 (3)º	Block, yellow
γ = 105.304 (3)º	0.38 × 0.31 × 0.18 mm
*V* = 944.1 (3) Å^3^	

### Data collection

**Table:**

Bruker SMART APEX CCD diffractometer	3504 independent reflections
Radiation source: fine-focus sealed tube	2695 reflections with *I* > 2σ(*I*)
Monochromator: graphite	*R*_int_ = 0.020
*T* = 298(2) K	θ_max_ = 25.7º
φ and ω scans	θ_min_ = 1.6º
Absorption correction: multi-scan(SADABS; Sheldrick, 1996)	*h* = −7→8
*T*_min_ = 0.912, *T*_max_ = 0.957	*k* = −12→12
5027 measured reflections	*l* = −17→17

### Refinement

**Table:**

Refinement on *F*^2^	Secondary atom site location: difference Fourier map
Least-squares matrix: full	Hydrogen site location: inferred from neighbouring sites
*R*[*F*^2^ > 2σ(*F*^2^)] = 0.057	H-atom parameters constrained
*wR*(*F*^2^) = 0.164	*w* = 1/[σ^2^(*F*_o_^2^) + (0.0858*P*)^2^ + 0.2952*P*] where *P* = (*F*_o_^2^ + 2*F*_c_^2^)/3
*S* = 1.05	(Δ/σ)_max_ = 0.002
3504 reflections	Δρ_max_ = 0.42 e Å^−^^3^
263 parameters	Δρ_min_ = −0.23 e Å^−^^3^
Primary atom site location: structure-invariant direct methods	Extinction correction: none

### Special details

**Table:**

Geometry. All e.s.d.'s (except the e.s.d. in the dihedral angle between two l.s. planes) are estimated using the full covariance matrix. The cell e.s.d.'s are taken into account individually in the estimation of e.s.d.'s in distances, angles and torsion angles; correlations between e.s.d.'s in cell parameters are only used when they are defined by crystal symmetry. An approximate (isotropic) treatment of cell e.s.d.'s is used for estimating e.s.d.'s involving l.s. planes.
Refinement. Refinement of *F*^2^ against ALL reflections. The weighted *R*-factor *wR* and goodness of fit *S* are based on *F*^2^, conventional *R*-factors *R* are based on *F*, with *F* set to zero for negative *F*^2^. The threshold expression of *F*^2^ > σ(*F*^2^) is used only for calculating *R*-factors(gt) *etc*. and is not relevant to the choice of reflections for refinement. *R*-factors based on *F*^2^ are statistically about twice as large as those based on *F*, and *R*- factors based on ALL data will be even larger.

### Fractional atomic coordinates and isotropic or equivalent isotropic displacement parameters (Å^2^)

**Table:**

	*x*	*y*	*z*	*U*_iso_*/*U*_eq_	
C1	0.2377 (5)	0.4244 (3)	0.6774 (2)	0.0530 (7)	
H1	0.2292	0.4775	0.7435	0.064*	
C2	0.2448 (5)	0.4829 (3)	0.6041 (3)	0.0613 (8)	
H2	0.2393	0.5744	0.6204	0.074*	
C3	0.2598 (5)	0.4057 (3)	0.5080 (2)	0.0546 (7)	
H3	0.2663	0.4450	0.4584	0.066*	
C4	0.2655 (4)	0.2661 (3)	0.4831 (2)	0.0430 (6)	
C5	0.2549 (4)	0.2104 (3)	0.55982 (18)	0.0374 (6)	
C6	0.2521 (3)	0.0678 (3)	0.53855 (18)	0.0349 (5)	
C7	0.2587 (4)	−0.0143 (3)	0.43867 (19)	0.0402 (6)	
C8	0.2705 (4)	0.0441 (3)	0.3616 (2)	0.0471 (7)	
H8	0.2750	−0.0120	0.2955	0.057*	
C9	0.2753 (4)	0.1789 (3)	0.3827 (2)	0.0485 (7)	
H9	0.2851	0.2153	0.3315	0.058*	
C10	0.2529 (4)	−0.1564 (3)	0.4190 (2)	0.0443 (6)	
H10	0.2592	−0.2151	0.3541	0.053*	
C11	0.2382 (4)	−0.2049 (3)	0.4948 (2)	0.0455 (6)	
H11	0.2333	−0.2976	0.4831	0.055*	
C12	0.2304 (4)	−0.1128 (3)	0.59278 (19)	0.0386 (6)	
C13	0.2012 (4)	−0.0886 (3)	0.7665 (2)	0.0490 (7)	
H13A	0.1173	−0.1480	0.7956	0.059*	
H13B	0.1426	−0.0160	0.7652	0.059*	
C14	0.4068 (4)	−0.0208 (3)	0.8319 (2)	0.0460 (7)	
C15	0.5178 (5)	−0.1004 (3)	0.8526 (2)	0.0557 (8)	
H15	0.4653	−0.1986	0.8265	0.067*	
C16	0.7067 (5)	−0.0345 (4)	0.9121 (3)	0.0692 (9)	
H16	0.7833	−0.0868	0.9270	0.083*	
C17	0.7788 (6)	0.1094 (4)	0.9488 (3)	0.0753 (10)	
H17	0.9061	0.1570	0.9890	0.090*	
C18	0.6621 (6)	0.1827 (4)	0.9257 (3)	0.0720 (10)	
H18	0.7132	0.2808	0.9511	0.086*	
C19	0.2308 (7)	0.3748 (4)	0.9157 (3)	0.0887 (12)	
H19A	0.2483	0.3543	0.9762	0.133*	
H19B	0.1107	0.4000	0.9102	0.133*	
H19C	0.3431	0.4521	0.9197	0.133*	
Cl1	0.20777 (12)	0.45705 (7)	0.21213 (5)	0.0541 (3)	
N1	0.2429 (3)	0.2941 (2)	0.65502 (16)	0.0419 (5)	
H4	0.2362	0.2694	0.7032	0.050*	
N2	0.2387 (3)	0.0189 (2)	0.61631 (15)	0.0377 (5)	
N3	0.4780 (4)	0.1207 (3)	0.86832 (19)	0.0580 (7)	
O1	0.2091 (3)	−0.17350 (18)	0.66282 (14)	0.0476 (5)	
O2	0.2322 (8)	0.5077 (4)	0.3177 (2)	0.1432 (16)	
O3	0.2709 (7)	0.5724 (3)	0.1857 (3)	0.1279 (13)	
O4	0.3128 (7)	0.3648 (4)	0.1769 (3)	0.1583 (17)	
O5	0.0081 (6)	0.3882 (4)	0.1659 (3)	0.1494 (16)	
O6	0.2165 (4)	0.2545 (2)	0.82875 (15)	0.0664 (6)	
H5	0.3072	0.2151	0.8417	0.100*	

### Atomic displacement parameters (Å^2^)

**Table:**

	*U*^11^	*U*^22^	*U*^33^	*U*^12^	*U*^13^	*U*^23^
C1	0.0681 (19)	0.0396 (14)	0.0503 (16)	0.0211 (14)	0.0085 (14)	0.0135 (12)
C2	0.080 (2)	0.0411 (15)	0.068 (2)	0.0208 (15)	0.0100 (17)	0.0261 (15)
C3	0.0631 (19)	0.0527 (17)	0.0588 (18)	0.0173 (15)	0.0088 (15)	0.0356 (15)
C4	0.0392 (14)	0.0474 (15)	0.0473 (15)	0.0123 (12)	0.0085 (12)	0.0248 (12)
C5	0.0343 (13)	0.0415 (13)	0.0373 (13)	0.0109 (11)	0.0062 (10)	0.0170 (11)
C6	0.0291 (12)	0.0387 (13)	0.0363 (13)	0.0085 (10)	0.0057 (10)	0.0156 (10)
C7	0.0315 (13)	0.0474 (14)	0.0397 (14)	0.0116 (11)	0.0057 (10)	0.0149 (11)
C8	0.0455 (15)	0.0615 (17)	0.0344 (13)	0.0174 (13)	0.0117 (11)	0.0169 (12)
C9	0.0496 (16)	0.0619 (18)	0.0435 (15)	0.0187 (14)	0.0125 (12)	0.0298 (13)
C10	0.0430 (15)	0.0437 (14)	0.0389 (14)	0.0149 (12)	0.0062 (11)	0.0064 (11)
C11	0.0461 (15)	0.0361 (13)	0.0488 (16)	0.0137 (12)	0.0051 (12)	0.0100 (12)
C12	0.0339 (13)	0.0379 (13)	0.0434 (14)	0.0105 (11)	0.0031 (11)	0.0162 (11)
C13	0.0567 (17)	0.0508 (16)	0.0479 (16)	0.0167 (14)	0.0156 (13)	0.0276 (13)
C14	0.0619 (18)	0.0460 (15)	0.0369 (14)	0.0193 (13)	0.0143 (12)	0.0211 (12)
C15	0.075 (2)	0.0495 (16)	0.0492 (17)	0.0266 (15)	0.0100 (15)	0.0215 (13)
C16	0.078 (2)	0.080 (2)	0.060 (2)	0.039 (2)	0.0054 (17)	0.0305 (18)
C17	0.072 (2)	0.082 (3)	0.061 (2)	0.019 (2)	−0.0055 (17)	0.0218 (19)
C18	0.085 (3)	0.0527 (18)	0.065 (2)	0.0110 (18)	−0.0040 (18)	0.0188 (16)
C19	0.124 (4)	0.088 (3)	0.057 (2)	0.051 (3)	0.022 (2)	0.0168 (19)
Cl1	0.0777 (6)	0.0433 (4)	0.0435 (4)	0.0249 (4)	0.0107 (3)	0.0149 (3)
N1	0.0504 (13)	0.0393 (11)	0.0388 (12)	0.0157 (10)	0.0074 (10)	0.0173 (9)
N2	0.0398 (12)	0.0377 (11)	0.0367 (11)	0.0126 (9)	0.0070 (9)	0.0152 (9)
N3	0.0745 (18)	0.0465 (14)	0.0524 (15)	0.0183 (13)	0.0056 (13)	0.0201 (11)
O1	0.0615 (12)	0.0371 (9)	0.0455 (11)	0.0141 (9)	0.0073 (9)	0.0189 (8)
O2	0.287 (5)	0.119 (3)	0.0502 (16)	0.101 (3)	0.037 (2)	0.0340 (17)
O3	0.204 (4)	0.087 (2)	0.108 (2)	0.035 (2)	0.043 (2)	0.0598 (19)
O4	0.217 (4)	0.144 (3)	0.135 (3)	0.143 (3)	0.026 (3)	0.015 (2)
O5	0.096 (3)	0.119 (3)	0.166 (4)	−0.003 (2)	−0.004 (2)	0.010 (3)
O6	0.0970 (17)	0.0684 (14)	0.0451 (12)	0.0420 (13)	0.0156 (11)	0.0226 (10)

### Geometric parameters (Å, °)

**Table:**

C1—N1	1.318 (3)	C13—O1	1.451 (3)
C1—C2	1.377 (4)	C13—C14	1.500 (4)
C1—H1	0.9300	C13—H13A	0.9700
C2—C3	1.357 (4)	C13—H13B	0.9700
C2—H2	0.9300	C14—N3	1.340 (4)
C3—C4	1.411 (4)	C14—C15	1.377 (4)
C3—H3	0.9300	C15—C16	1.375 (5)
C4—C5	1.402 (4)	C15—H15	0.9300
C4—C9	1.426 (4)	C16—C17	1.362 (5)
C5—N1	1.361 (3)	C16—H16	0.9300
C5—C6	1.431 (3)	C17—C18	1.363 (5)
C6—N2	1.368 (3)	C17—H17	0.9300
C6—C7	1.397 (3)	C18—N3	1.337 (4)
C7—C10	1.423 (4)	C18—H18	0.9300
C7—C8	1.425 (4)	C19—O6	1.410 (4)
C8—C9	1.346 (4)	C19—H19A	0.9600
C8—H8	0.9300	C19—H19B	0.9600
C9—H9	0.9300	C19—H19C	0.9600
C10—C11	1.340 (4)	Cl1—O4	1.368 (3)
C10—H10	0.9300	Cl1—O3	1.375 (3)
C11—C12	1.413 (4)	Cl1—O2	1.383 (3)
C11—H11	0.9300	Cl1—O5	1.388 (4)
C12—N2	1.303 (3)	N1—H4	0.8111
C12—O1	1.354 (3)	O6—H5	0.8900
			
N1—C1—C2	120.9 (3)	C14—C13—H13A	109.5
N1—C1—H1	119.6	O1—C13—H13B	109.5
C2—C1—H1	119.6	C14—C13—H13B	109.5
C3—C2—C1	119.4 (3)	H13A—C13—H13B	108.1
C3—C2—H2	120.3	N3—C14—C15	121.6 (3)
C1—C2—H2	120.3	N3—C14—C13	116.9 (3)
C2—C3—C4	120.3 (3)	C15—C14—C13	121.5 (3)
C2—C3—H3	119.9	C16—C15—C14	119.8 (3)
C4—C3—H3	119.9	C16—C15—H15	120.1
C5—C4—C3	118.2 (2)	C14—C15—H15	120.1
C5—C4—C9	119.1 (2)	C17—C16—C15	118.4 (3)
C3—C4—C9	122.7 (2)	C17—C16—H16	120.8
N1—C5—C4	118.6 (2)	C15—C16—H16	120.8
N1—C5—C6	120.4 (2)	C16—C17—C18	119.2 (3)
C4—C5—C6	121.0 (2)	C16—C17—H17	120.4
N2—C6—C7	123.9 (2)	C18—C17—H17	120.4
N2—C6—C5	118.3 (2)	N3—C18—C17	123.4 (3)
C7—C6—C5	117.8 (2)	N3—C18—H18	118.3
C6—C7—C10	116.8 (2)	C17—C18—H18	118.3
C6—C7—C8	120.5 (2)	O6—C19—H19A	109.5
C10—C7—C8	122.7 (2)	O6—C19—H19B	109.5
C9—C8—C7	121.2 (2)	H19A—C19—H19B	109.5
C9—C8—H8	119.4	O6—C19—H19C	109.5
C7—C8—H8	119.4	H19A—C19—H19C	109.5
C8—C9—C4	120.4 (2)	H19B—C19—H19C	109.5
C8—C9—H9	119.8	O4—Cl1—O3	111.0 (3)
C4—C9—H9	119.8	O4—Cl1—O2	111.7 (2)
C11—C10—C7	119.4 (2)	O3—Cl1—O2	107.0 (2)
C11—C10—H10	120.3	O4—Cl1—O5	108.2 (3)
C7—C10—H10	120.3	O3—Cl1—O5	107.6 (3)
C10—C11—C12	119.0 (2)	O2—Cl1—O5	111.2 (3)
C10—C11—H11	120.5	C1—N1—C5	122.6 (2)
C12—C11—H11	120.5	C1—N1—H4	113.1
N2—C12—O1	121.1 (2)	C5—N1—H4	124.3
N2—C12—C11	124.7 (2)	C12—N2—C6	116.2 (2)
O1—C12—C11	114.2 (2)	C18—N3—C14	117.6 (3)
O1—C13—C14	110.5 (2)	C12—O1—C13	119.09 (19)
O1—C13—H13A	109.5	C19—O6—H5	109.6
			
N1—C1—C2—C3	−0.8 (5)	C7—C10—C11—C12	−0.5 (4)
C1—C2—C3—C4	0.8 (5)	C10—C11—C12—N2	−0.8 (4)
C2—C3—C4—C5	0.1 (4)	C10—C11—C12—O1	177.9 (2)
C2—C3—C4—C9	178.2 (3)	O1—C13—C14—N3	115.5 (3)
C3—C4—C5—N1	−1.1 (4)	O1—C13—C14—C15	−63.9 (3)
C9—C4—C5—N1	−179.2 (2)	N3—C14—C15—C16	0.0 (4)
C3—C4—C5—C6	177.5 (2)	C13—C14—C15—C16	179.3 (3)
C9—C4—C5—C6	−0.6 (4)	C14—C15—C16—C17	−0.2 (5)
N1—C5—C6—N2	−0.5 (3)	C15—C16—C17—C18	0.2 (5)
C4—C5—C6—N2	−179.1 (2)	C16—C17—C18—N3	0.0 (6)
N1—C5—C6—C7	178.1 (2)	C2—C1—N1—C5	−0.1 (4)
C4—C5—C6—C7	−0.5 (4)	C4—C5—N1—C1	1.1 (4)
N2—C6—C7—C10	−0.6 (4)	C6—C5—N1—C1	−177.5 (2)
C5—C6—C7—C10	−179.1 (2)	O1—C12—N2—C6	−177.3 (2)
N2—C6—C7—C8	179.3 (2)	C11—C12—N2—C6	1.3 (4)
C5—C6—C7—C8	0.8 (4)	C7—C6—N2—C12	−0.6 (3)
C6—C7—C8—C9	−0.1 (4)	C5—C6—N2—C12	178.0 (2)
C10—C7—C8—C9	179.9 (3)	C17—C18—N3—C14	−0.2 (5)
C7—C8—C9—C4	−1.0 (4)	C15—C14—N3—C18	0.2 (4)
C5—C4—C9—C8	1.3 (4)	C13—C14—N3—C18	−179.2 (3)
C3—C4—C9—C8	−176.7 (3)	N2—C12—O1—C13	−1.8 (3)
C6—C7—C10—C11	1.1 (4)	C11—C12—O1—C13	179.4 (2)
C8—C7—C10—C11	−178.8 (2)	C14—C13—O1—C12	−90.4 (3)

### Hydrogen-bond geometry (Å, °)

**Table:**

*D*—H···*A*	*D*—H	H···*A*	*D*···*A*	*D*—H···*A*
N1—H4···O6	0.81	1.85	2.648 (3)	167
O6—H5···N3	0.89	1.85	2.738 (4)	175
C3—H3···O2	0.93	2.32	3.238 (5)	170
C13—H13B···O6	0.97	2.58	3.405 (4)	143
C8—H8···Cg3^i^	0.93	2.81	3.650 (2)	151

Symmetry codes:  (i) −*x*+1, −*y*, −*z*+1.
